# A Prospective Epidemiological Study of Acute Mountain Sickness in Nepalese Pilgrims Ascending to High Altitude (4380 m)

**DOI:** 10.1371/journal.pone.0075644

**Published:** 2013-10-09

**Authors:** Martin J. MacInnis, Eric A. Carter, Michael G. Freeman, Bidur Prasad Pandit, Ashmita Siwakoti, Ankita Subedi, Utsav Timalsina, Nadia Widmer, Ghan Bahadur Thapa, Michael S. Koehle, Jim L. Rupert

**Affiliations:** 1 School of Kinesiology, University of British Columbia, Vancouver, Canada; 2 Faculty of Medicine, University of Glasgow, Glasgow, Scotland; 3 Maharajgunj Medical Campus, Institute of Medicine, Tribhuvan University, Nepal; 4 Faculty of Medicine, University of British Columbia, Vancouver, Canada; University of California Los Angeles, United States of America

## Abstract

**Background:**

Each year, thousands of pilgrims travel to the Janai Purnima festival in Gosainkunda, Nepal (4380 m), ascending rapidly and often without the aid of pharmaceutical prophylaxis.

**Methods:**

During the 2012 Janai Purnima festival, 538 subjects were recruited in Dhunche (1950 m) before ascending to Gosainkunda. Through interviews, subjects provided demographic information, ratings of AMS symptoms (Lake Louise Scores; LLS), ascent profiles, and strategies for prophylaxis.

**Results:**

In the 491 subjects (91% follow-up rate) who were assessed upon arrival at Gosainkunda, the incidence of AMS was 34.0%. AMS was more common in females than in males (RR = 1.57; 95% CI = 1.23, 2.00), and the AMS incidence was greater in subjects >35 years compared to subjects ≤35 years (RR = 1.63; 95% CI = 1.36, 1.95). There was a greater incidence of AMS in subjects who chose to use garlic as a prophylactic compared to those who did not (RR = 1.69; 95% CI = 1.26, 2.28). Although the LLS of brothers had a moderate correlation (intraclass correlation = 0.40, p = 0.023), sibling AMS status was a weak predictor of AMS.

**Conclusions:**

The incidence of AMS upon reaching 4380 m was 34% in a large population of Nepalese pilgrims. Sex, age, and ascent rate were significant factors in the development of AMS, and traditional Nepalese remedies were ineffective in the prevention of AMS.

## Introduction

Failure to acclimatise upon ascent to altitudes above 2500 m manifests as acute mountain sickness (AMS), an illness characterized by headache, nausea, dizziness, fatigue, and poor sleep quality [1]. The symptoms of AMS are usually mild and self-limiting, but symptoms can become incapacitating [2]. In some cases, AMS may even progress to high-altitude cerebral edema, a rare but life-threatening condition [3]. Annually, AMS affects millions of high-altitude sojourners [4], impacting their health, travel, and economic productivity.

Ascent profile and individual characteristics determine one’s likelihood of developing AMS. Strong positive correlations exist between the incidence of AMS and both the altitude attained and the rate of ascent [3]. Yet, for a given ascent regimen, individuals differ greatly in terms of their susceptibilities to AMS, with some developing AMS and others acclimatising well to hypoxia. The basis of these individual differences in susceptibility to AMS is not well understood (reviewed in Ref. [5]), and little progress has been made in establishing predictive tools for AMS.

The incidence of AMS might be reduced if the most susceptible individuals were identified pre-ascent and provided with precautionary advice [6]. Several physiological variables have been tested for the potential to predict AMS susceptibility (*e.g.*, chemosensitivity, heart rate variability), but most variables (excluding perhaps those variables measured as part of Richalet’s hypoxic exercise test [7]) are not sufficiently reliable and many procedures are not feasible in all settings and populations, especially regions with limited medical resources. Demographic factors and family history of AMS may provide a simpler assessment of an individual’s susceptibility; however, these factors must be strongly associated with the incidence of AMS if they are to be useful predictors of AMS. Furthermore, risk factors previously identified in studies of tourists [8]–[10], mountaineers [11], [12], and high-altitude laborers and soldiers [13], [14] must be re-assessed in unspecialized populations to ensure that these results can be generalized.

Janai Purnima is an annual religious festival occurring in the Nepalese Himalaya on the full moon of Shrawan/Bhadra (the forth/fifth month in the Nepali calendar). During the festival, thousands of pilgrims ascend rapidly (*i.e.*, in 1–2 days) from Dhunche (1950 m) to Gosainkunda (4380 m). The goals of this study were (1) to ascertain the incidence of AMS in a large population of Nepalese pilgrims at high altitude and (2) to investigate factors related to AMS susceptibility in this general population.

## Methods

### Overview

This study used a prospective, longitudinal design: subjects were recruited in Dhunche (1950 m) over a 5-day period preceding the 2012 Janai Purnima festival and assessed in Gosainkunda (4380 m) over a similar period of time. Interested subjects were able to provide verbal or written informed consent. Based on previous experiences at this location, verbal consent was the preference of most pilgrims. While verbal consent was not documented, data were not collected until informed consent was obtained. The University of British Columbia Clinical Research Ethics Board and the Nepal Health Research Council granted ethical approval for this study (including verbal consent), and the district health, public health, and chief district officers of Rasuwa provided regional approval.

### Recruitment

Most pilgrims traveled to Dhunche from the Kathmandu Valley (∼1400 m) by motor vehicle (*e.g.*, bus or motorcycle) and by foot. In Dhunche, the main street in the village was canvassed to recruit subjects. Potential subjects were excluded if they had spent any time above 2500 m in the two months prior to the study. A Nepalese medical student or intern conducted an interview with each subject to collect demographic data (*i.e.*, age, sex), family information (*i.e.*, name, relatives participating in study), and a baseline Lake Louise Score (LLS). Subjects wore numbered paper bracelets on their wrists for identification at the high-altitude site.

### Assessment

**Figure 1:**
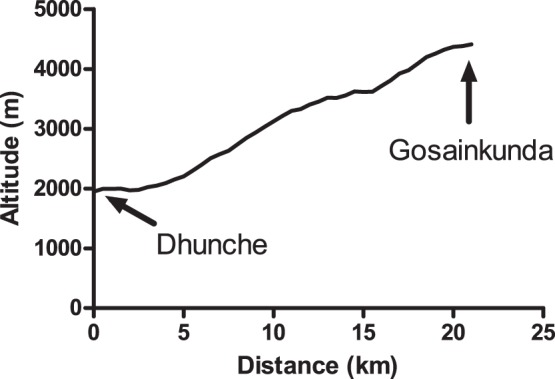
The ascent profile from Dhunche (1950 m) to Gosainkunda (4380 m). All subjects ascended to Gosainkunda via this route in 1, 2, or 3 days. The average grade of the ascent was approximately 12%.

All subjects traveled on the same trail from Dhunche to Gosainkunda (Figure 1), but ascent rates were self-selected. Subjects were assessed upon arrival to Gosainkunda, and those who stayed over night were asked to report the next morning for a follow-up assessment. At both time points, a Nepalese medical student or intern administered the LLS questionnaire [1] in Nepali under the supervision of an experienced physician. Subjects with a LLS ≥3 (including a headache score ≥1) were considered to be positive for AMS (AMS+), while subjects without a headache or with a LLS <3 were considered to be negative for AMS (AMS-). Subjects also provided information about their ascent profiles (number of days and sleeping locations), strategies for prophylaxis, general health, and current medications.

### Statistical Analysis

Continuous data are presented as means and (standard deviations) and categorical data are presented as counts and/or percentages. A p-value less than 0.05 was considered statistically significant for all statistical tests. Independent t-tests were used for continuous variables, and Chi-squared (X^2^) tests were used categorical variables. Risk ratios (RR) were calculated to interpret the strength of associations between categorical variables and the incidence of AMS. Multiple logistic regression was performed to interpret the combined effect of individually significant categorical variables.

For siblings and parent-offspring pairs, the first subject to be enrolled in the study was regarded as the proband. The relative risk ratio (the risk in siblings of affected probands relative to the risk in siblings of unaffected probands) and the recurrence risk ratio (the risk in siblings of affected probands relative to the population risk) were calculated for siblings. The agreement among the LLS of siblings and parent-offspring pairs was determined using one-way random effects intraclass correlations (ICC).

## Results

### Subject Characteristics and AMS Incidence

A total of 538 subjects recruited in Dhunche were included in the study. Baseline LLS were low in all subjects (97% of subjects were below a LLS of 2). Of those initially recruited, 501 (93%) presented for follow-up at Gosainkunda (37 subjects (7%) were lost to follow-up; Table 1). Ten of these subjects were not assessed upon arrival to Gosainkunda (*i.e,* they were assessed the morning after arrival), and they were excluded from further analyses. Dropouts and retained subjects had similar ages (35.6 (12.9) and 36.8 (13.2) years, respectively; t = −0.521; p = 0.60), but dropouts were more likely to be male (92% vs. 69%; X^2^ = 8.07; p = 0.005) and more likely to have traveled to Gosainkunda previously (32% vs. 17%; X^2^ = 5.21; p = 0.022).

**Table 1:** Characteristics of Nepalese pilgrims assessed upon arrival to Gosainkunda (4380 m).

Variable	Arrival dataset
Sample size	491
Age (years)	36.7 (13.2)
Sex (% male)	70.1
Smoking history (% yes)	29.3
First trek to Gosainkunda? (% yes)	82.7
Ascent rate (days above 3000 m)^#^	1.9 (0.48)
Sleeping altitude of previous night (m)	3566 (652)
Lake Louise Score	2.5 (2.0)

Data are presented as mean (standard deviation) or as a percent.

Ninety-one percent (n = 491) of subjects were assessed upon arrival to Gosainkunda, and a group of these subjects (n = 125; 25.5%) was assessed a second time the morning after arrival. Subjects assessed at both time points were similar to subjects only assessed upon arrival based on age, sex, and LLS (on arrival; data not shown). Analyses of risk factors, family data, and prophylaxis are based only on data collected upon arrival.

**Figure 2:**
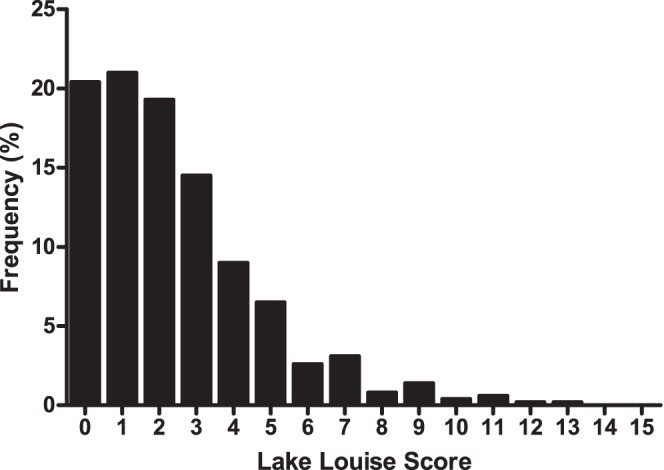
The frequency of Lake Louise Scores in Nepalese pilgrims (n = 491) upon arrival to Gosainkunda (4380 m).

**Figure 3:**
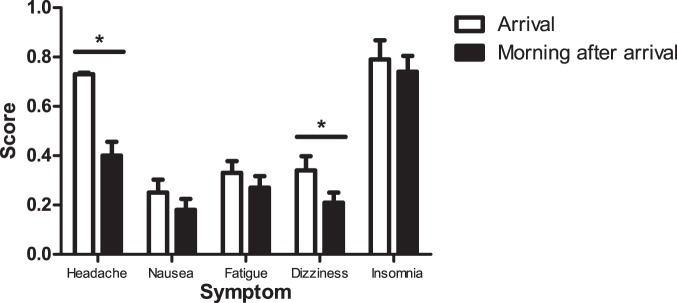
A comparison of the AMS symptoms of Nepalese pilgrims who were assessed upon arrival to, and on the morning after arrival to, Gosainkunda (n = 125). Headache and dizziness scores were significantly lower the morning after arrival (t = 4.46; p<0.001; t = 2.33; p = 0.02, respectively). Error bars represent one standard error of the mean.

Subject characteristics are presented in Table 1. The majority of subjects (n = 366) trekked to Gosainkunda in 2 days (others ascended in 1 or 3 days). The incidence of AMS upon arrival to Gosainkunda was 34.0%, and the distribution of LLS is displayed in Figure 2. For those subjects assessed at both time points, the incidence of AMS decreased from 40.0% on arrival to 20.0% on the morning after arrival (X^2^ = 11.7; p = 0.001), and the LLS was significantly lower in the morning (1.8 (2.0)) than on arrival (2.5 (2.0); t = 3.42; p<0.001), largely due to decreases in the severity of headache and dizziness symptoms (Figure 3).

### Individual Risk Factors

Age was divided at the median (≤35 years and >35 years) to create a dichotomous variable. The 3-day ascent group was removed from the ascent-rate analysis because this group was much smaller (n = 27) and significantly older than the two groups that ascended more rapidly (data not shown).

Age positively correlated with LLS (rho = 0.251; p<0.001), and age was the single strongest predictor of AMS: subjects >35 years were 63% more likely to develop AMS than subjects ≤35 years of age (Table 2). Females (57%) and subjects ascending in 1 day (37%) were more likely to develop AMS than males and subjects ascending in 2 days, respectively (Table 2). Smoking status and previous travel to Gosainkunda were not significantly associated with the incidence of AMS (Table 2); however, the incidence of AMS was ∼11% lower in smokers than in non-smokers and ∼8% lower in subjects who previously visited Gosainkunda compared to first-time travelers.

**Table 2:** Statistical relationships between dichotomous variables and the incidence of acute mountain sickness (AMS) in Nepalese pilgrims upon arrival to Gosainkunda (4380 m).

Category	Subcategory	Sample size (n (%))		X^2^		Relative Risk	
		Total	AMS+	Statistic	p-value	Statistic	95% CI
**Sex**	**Male**	344 (70.1)	100 (29.1)				
	**Female**	147 (29.9)	67 (45.5)	12.5*	<0.001	1.57*	1.23, 2.00
**Age** †	**≤35 years**	261 (53.5)	63 (24.1)				
	**>35 years**	227 (46.5)	104 (45.8)	25.3*	<0.001	1.63*	1.36, 1.95
**Smoking**	**Yes**	147 (29.9)	42 (25.1)				
	**No**	344 (70.1)	125 (36.3)	2.77	0.096	1.27	0.95, 1.70
**Ascent** ‡	**2 days**	366 (80.3)	113 (30.9)				
	**1 day**	90 (19.7)	38 (42.2)	4.20*	0.040	1.37*	1.03, 1.82
**First ascent to Gosainkunda?**	**No**	85 (17.3)	23 (27.1)				
	**Yes**	406 (82.7)	144 (35.5)	2.21	0.137	1.31	0.90, 1.90

† The age indicator was missing 3 values due to incomplete data forms.

‡ The 3-day ascent group was removed from the analysis of ascent rate data (see text).

* This result is statistically significant (*i.e.*, p<0.05).

Despite the lack of independence among the significant predictor variables (sex, age, ascent rate; data not shown), each variable was a significant predictor of AMS in a multiple logistic regression equation (Table 3), suggesting that the effects of each variable persisted when the other variables were controlled.

**Table 3:** The results of multiple binary logistic regression for the individual predictors of AMS in Nepalese pilgrims upon arrival to Gosainkunda (4380 m).

Variable†	B (SE)	Odds ratio		p-value
		Statistic	95% CI	
Constant	−1.57 (0.19)	0.60		0.003
Sex	0.57 (0.23)	1.77*	1.14, 2.75	0.011
Age	1.06 (0.22)	2.89*	1.89, 4.39	<0.001
Ascent rate	0.81 (0.17)	2.26*	1.35, 3.76	0.002

† Males, subjects ≤35 years, and the 2-day ascent group were used as the reference categories for the calculation of odds ratios.

* This result is significant (*i.e.*, p<0.05).

### Prophylaxis

Pharmaceutical prophylaxis was uncommon (acetazolamide: 7%; paracetamol: 7%; non-steroidal anti-inflammatory drugs (NSAID): 3%) and not associated with a decreased occurrence of AMS (Table 4); however, many pilgrims (70%) ingested one or more of the following foods to prevent AMS: garlic, ginger, lemon, or mountain pepper (*Zanthoxylum* sp.). Of these foods, garlic and mountain pepper were associated with a greater risk of AMS (Table 4). Only the deliberate ingestion of these foods to prevent AMS was recorded (*i.e.,* subjects were not asked about their diets), and details of dosage and timing were not obtained for pharmaceutical or dietary prophylaxis.

**Table 4:** Statistical relationships between the use of pharmaceutical and dietary prophylaxis and the incidence of acute mountain sickness (AMS) in Nepalese pilgrims upon arrival to Gosainkunda (4380 m).

Prophylactic agent	Use	Sample size (n (%))		X^2^		Relative Risk	
		Total	AMS+	Statistic	p-value	Statistic	95% CI
**Acetazolamide**	**No**	457	155 (33.9)				
	**Yes**	34	12 (35.3)	0.027	0.87	1.04	0.65, 1.67
**Paracetamol**	**No**	459	154 (33.6)				
	**Yes**	32	13 (40.6)	0.67	0.41	1.21	0.78, 1.88
**NSAID**	**No**	478	162 (33.9)				
	**Yes**	13	5 (38.5)	0.12	0.73	1.14	0.56, 2.28
**Garlic**	**No**	178	42 (23.6)				
	**Yes**	313	125 (39.9)	13.50*	<0.001	1.69*	1.26, 2.28
**Ginger**	**No**	371	120 (32.3)				
	**Yes**	120	47 (39.2)	1.88	0.17	1.21	0.93, 1.58
**Lemon**	**No**	366	117 (31.5)				
	**Yes**	125	50 (40.0)	2.68	0.10	1.25	0.96, 1.63
**Mountain pepper**	**No**	448	147 (32.8)				
	**Yes**	43	20 (47.6)	3.28	0.07	1.42*	1.00, 2.1

* This result is statistically significant (*i.e.*, p<0.05).

### Family Data

Forty-eight pairs of siblings were identified in the dataset. The relative risk ratio was 1.38 (95% CI = 0.68–2.83), and the sibling recurrence risk ratio was 1.47 (95% CI = 0.82–2.62), but neither ratio was significantly different from 1.0 (p>0.05). The ICC was significant for the LLS of brothers (0.40; p = 0.023) but not for all siblings (0.16; p = 0.13). Probands (36.8 (12.9) years) and their siblings (38.0 (13.1) years) were of similar ages (t = −0.421; p = 0.67).

Twenty-nine parent-offspring pairs were identified in the dataset, but there was no relationship between the LLS of parents and their offspring (ICC = 0.12; p = 0.26). Ten parents developed AMS compared to only five offspring who developed AMS, but parents (54.4 (10.7) years) were significantly older than their offspring (31.7 (12.0) years; t = 7.48; p<0.001).

## Discussion

To our knowledge, this is the largest study of AMS in pilgrims traveling to high-altitude. Our main findings were (1) sex, age, and ascent rate were significant risk factors for the development of AMS upon arrival to 4380 m; (2) the use of traditional preventatives (*i.e.,* garlic and mountain pepper) was associated with an increased risk of AMS; and (3) sibling AMS status was a weak predictor of AMS, despite a moderate correlation in the LLS of brothers.

Ninety-one percent of subjects who were recruited in Dhunche completed the study. This completion rate likely reflects the religious devotion of the pilgrims, who are known to carry out their religious duties despite significant hardship and AMS symptoms. The few subjects who dropped out of the study may have descended for health reasons (*e.g.,* altitude illness), but even if all of those subjects lost to follow-up were attributed to the AMS+ group, that hypothetical AMS incidence (38%) would not be much greater than our reported incidence.

Previous studies at the Janai Purnima festival in Gosainkunda reported AMS incidences of 5% [15] and 68% [16] in Nepalese pilgrims. The former study may have underestimated the true incidence of AMS at the festival because only visibly ill pilgrims were counted as having AMS, while all other passersby were assumed to be free of AMS. The latter study used a random sampling protocol at the high-altitude site, and the incidence might have been greater as a consequence of the cross-sectional design. The AMS incidence reported in this study is similar to incidences measured at different locations with comparable altitudes (*e.g.,* Ref. [8], [17]).

The severity of AMS is reportedly greater after sleeping at a new altitude than upon arrival [18]; however, the subjects in this study who were assessed upon arrival and the morning after arrival were symptomatically better the morning after arriving at Gosainkunda. While those subjects who presented for the morning-after-arrival assessment were similar to those who did not present (based on demographic and ascent data collected upon arrival), we cannot rule out the possibility that pilgrims who awoke feeling unwell chose to forego the morning assessment. If this scenario were true, the morning-after-arrival dataset could be biased with respect to LLS (*i.e.,* a lower incidence and severity of AMS would be expected), and this aspect of our study should be interpreted cautiously. Alternatively, these subjects may have had sufficient time to acclimatise, given that the night spent at Gosainkunda was the second night at an altitude above 3000 m for 57% of subjects. Improvements in LLS were largely the result of decreased headache and dizziness scores, but the sleep quality score did not improve. In studies at 4559 m, poor subjective ratings of sleep quality persisted for days, despite decreases in the LLS [19], [20].

Similar to earlier studies at the Janai Purnima festival (Ref. [16] and M. Freeman, unpublished data), the incidence of AMS was significantly greater in females than males. This finding is supported by a large prospective study [7] and several smaller studies [9], [21], but not all studies reported sex as a risk factor for AMS (*e.g.*, Ref. [10], [11], [22], [23]). Given the agreement among studies of Nepalese women, sex seems to be a strong risk factor for AMS in this population. One reason for the sex difference could be cultural, with females possibly more likely to admit AMS than males [16]. Alternatively, females may have been more likely to avoid food and drink (for religious reasons) than men. Hypoglycemia and dehydration can be confused with AMS, as the symptoms of both conditions overlap considerably [3], [24].

The severity and incidence of AMS increased with age. This finding is contrary to many previous studies, including a large prospective study [7] and several smaller studies [21], [23]. The relationship between age and AMS reported in our study might be partly due to the religious nature of the Janai Purnima festival: the older pilgrims may have become more dehydrated than the younger pilgrims by avoiding food and drink on the ascent [16]. Alternatively, there may be differences (*e.g.*, health and socioeconomic status) in individuals who travel to altitude for recreation and pilgrims who feel compelled to travel to altitude for religious purposes. Specifically, older pilgrims likely had significant co-morbidities (*e.g.* neurological disorders and pulmonary diseases) that could increase their risk of AMS [25], [26], and many pilgrims believe that blessings from Lord Shiva can cure these ailments. In studies of mountaineering populations, individuals who are susceptible to AMS may be less likely to continue mountaineering, possibly making older mountaineers a self-selected group that is relatively resistant to AMS. This process may partially account for the previously reported lower incidence of AMS in older individuals.

As expected, pilgrims who ascended to Gosainkunda from below 3000 m in one day were more likely to develop AMS than those who ascended in two days, which is likely a result of insufficient time to acclimatize [10]. Because subjects chose their own ascent rates, we cannot rule out factors that may have been related to this choice; however, the relationship between ascent rate and AMS incidence is supported by the literature [10], [22].

Two recent studies reported that smokers were less likely to develop AMS than non-smokers [27], [28], and, although not statistically significant, our results support these findings. Smoking had a relatively small effect on the incidence and severity of AMS, and it is not recommended for the prevention of AMS for many reasons, including its deleterious effects on overall health, exercise capacity, and frostbite susceptibility [28].

The rate of pharmaceutical prophylaxis amongst Nepalese pilgrims is typically low, although it may be increasing, as the proportion of pilgrims taking acetazolamide in this study was 2.5 times higher than in a previous report [16]. Those who chose to take pharmaceuticals had a similar incidence compared to those who did not, which strongly disagrees with multiple studies [29], [30] and a recent meta-analysis [31]; however, our study was not designed to test the effectiveness of pharmaceutical prophylaxis, and the lack of effect may be due to inappropriate dosages and strategies or confounding factors associated with a non-random design.

The use of traditional AMS preventatives (*i.e.*, garlic and mountain pepper) was associated with a higher incidence of AMS relative to those who did not consume these foods with the purpose of preventing AMS. It is not likely that these preventatives caused AMS through a physiological pathway, as all subjects likely ingested some amount of each food through their regular diets. While studies have not tested the effects of garlic on AMS, the ingestion of garlic prior to hypoxic exercise did not affect oxygen consumption, oxygen saturation, heart rate, blood pressure, or exercise performance relative to placebo [32]. Pilgrims who consumed foods that they expected to prevent AMS might have ignored other means of AMS prophylaxis (*e.g.*, slow ascent rate), increasing the probability that they would experience AMS. Until a randomized control trial can demonstrate that these foods prevent AMS, Nepalese pilgrims (and others) should be discouraged from relying solely on traditional Nepalese methods as a means to prevent AMS. Abstention from these foods is not likely necessary while ascending to high altitude.

Family history was not a significant risk factor for AMS in this population, but signs of familial aggregation were still evident. Sibling, parent and offspring AMS status were weak predictors of AMS. The LLS of brothers had a moderate correlation, but correlations for the LLS of other sibling pairs and parent-offspring pairs were weak. Differences in age and sex may have contributed to the weak correlations. Establishing whether or not AMS aggregates in families is essential for genetic studies of AMS susceptibility, as familial aggregation is a criterion for the genetic basis of AMS [5]. Until larger family studies are conducted, family history of AMS will not be useful for counseling travelers to high altitude.

Our data collection was limited by the austere environment of Gosainkunda (*e.g.,* no electricity) and the short amount of time we had with each pilgrim. We were unable to control for several variables that could be related to AMS susceptibility, such as underlying health conditions. There has been significant interest in the utility of pulse oximetry in the diagnosis of AMS [33], and we previously demonstrated that pulse oximetry was associated with AMS status at this particular field site [34]; however, because of the inclement weather, limited indoor facilities, and large number of subjects who visited our site in a short period of time, we were not able to accurately record heart rate and oxygen saturation data from all subjects. These data are included as a supplemental file (Table S1), but the data may not be true resting values because of the testing environment.

## Conclusions

In a large prospective study of Nepalese pilgrims, we observed an AMS incidence (34.0%) after a rapid ascent from 1950 m to 4380 m. Females and older pilgrims were more likely to develop AMS than male and younger pilgrims, and ascent rate was a risk factor for AMS. The ineffectiveness of dietary supplements suggests that traditional preventative strategies (*e.g.*, garlic) should not be relied on as the sole means of AMS prevention. Weak to moderate relationships were identified between the LLS and AMS status of siblings and parent-offspring pairs, suggesting a modest role for genetics in this cohort.

## Supporting Information

Table S1 The mean (standard deviation) oxygen saturation (S_P_O_2_) and heart rate (HR) of subjects with (AMS+) and without (AMS–) acute mountain sickness (AMS).
(DOCX)
