# Supplementary material for: A Prospective Epidemiological Study of Acute Mountain Sickness in Nepalese Pilgrims Ascending to High Altitude (4380 m)
Source: PLoS One. 2013 Oct 9;8(10):e75644. doi: 10.1371/journal.pone.0075644 (PMC3794000; doi:10.1371/journal.pone.0075644)
Supplement: Table S1 — The mean (standard deviation) oxygen saturation (SPO2) and heart rate (HR) of subjects with (AMS+) and without (AMS–) acute mountain sickness (AMS). (DOCX) [file pone.0075644.s001.docx]

Table S1. The mean (standard deviation) oxygen saturation (S_P_O_2_) and heart rate (HR) of subjects with (AMS+) and without (AMS-) acute mountain sickness (AMS).

| **Variable** | **Sample size**  **(AMS+: AMS-)** | **AMS+** | **AMS-** | **p-value** |
| --- | --- | --- | --- | --- |
| S_P_O_2_ (%) | 322:167 | 81.1 (5.5) | 82.1 (5.0) | 0.07 |
| HR (bpm) | 322:167 | 106 (15) | 105 (15) | 0.33 |

bpm, beats per minute.

Note: S_P_O_2_ and HR were measured using a portable finger pulse oximeter (GO_2_; Nonin Medical Inc.; Minneapolis, MN).
